# Alterations in GABA_A_-Receptor Trafficking and Synaptic Dysfunction in Brain Disorders

**DOI:** 10.3389/fncel.2019.00077

**Published:** 2019-03-07

**Authors:** Miranda Mele, Rui O. Costa, Carlos B. Duarte

**Affiliations:** ^1^CNC—Center for Neuroscience and Cell Biology, University of Coimbra, Coimbra, Portugal; ^2^Institute for Interdisciplinary Research, University of Coimbra, Coimbra, Portugal; ^3^Department of Life Sciences, University of Coimbra, Coimbra, Portugal

**Keywords:** GABA_A_ receptor trafficking, epilepsy, brain ischemia, Alzheimer’s disease, Huntington’s disease, Parkinson’s disease

## Abstract

GABA_A_ receptors (GABA_A_R) are the major players in fast inhibitory neurotransmission in the central nervous system (CNS). Regulation of GABA_A_R trafficking and the control of their surface expression play important roles in the modulation of the strength of synaptic inhibition. Different pieces of evidence show that alterations in the surface distribution of GABA_A_R and dysregulation of their turnover impair the activity of inhibitory synapses. A diminished efficacy of inhibitory neurotransmission affects the excitatory/inhibitory balance and is a common feature of various disorders of the CNS characterized by an increased excitability of neuronal networks. The synaptic pool of GABA_A_R is mainly controlled through regulation of internalization, recycling and lateral diffusion of the receptors. Under physiological condition these mechanisms are finely coordinated to define the strength of GABAergic synapses. In this review article, we focus on the alteration in GABA_A_R trafficking with an impact on the function of inhibitory synapses in various disorders of the CNS. In particular we discuss how similar molecular mechanisms affecting the synaptic distribution of GABA_A_R and consequently the excitatory/inhibitory balance may be associated with a wide diversity of pathologies of the CNS, from psychiatric disorders to acute alterations leading to neuronal death. A better understanding of the cellular and molecular mechanisms that contribute to the impairment of GABAergic neurotransmission in these disorders, in particular the alterations in GABA_A_R trafficking and surface distribution, may lead to the identification of new pharmacological targets and to the development of novel therapeutic strategies.

## Introduction

The appropriate equilibrium between excitatory and inhibitory neurotransmission, which is mainly mediated by glutamate and γ-aminobutyric acid (GABA), respectively, is necessary for the correct function of neuronal circuits in the central nervous system (CNS; Smith and Kittler, 2010). Therefore, the control of GABAergic synaptic strength and transmission plays a crucial role in the maintenance of the excitatory/inhibitory synaptic balance (Smith and Kittler, 2010; Mele et al., 2016). An impairment of these mechanisms leading to neuronal hyperexcitability is a common and early event that characterizes several brain disorders (McCormick and Contreras, 2001; Saxena and Caroni, 2011).

The neurotransmitter GABA acts, in part, through activation of GABA_A_ receptors (GABA_A_R), which are heteropentameric chloride channels, composed in most cases of 2α-, 2β-, and 1γ2-subunits (Rudolph and Möhler, 2004). GABA_A_R with different subunit compositions have different physiological and pharmacological properties, are differentially expressed throughout the brain and are targeted to different subcellular regions (Fritschy and Mohler, 1995; Nusser et al., 1998b). Receptors composed of α1, α2 or α3 subunits together with β and γ subunits are benzodiazepine-sensitive and largely synaptically located, mediating most phasic inhibition in the brain (Rudolph and Möhler, 2004). The synaptic localization of these receptors is determined by the direct interaction of the alpha subunits with the scaffold protein gephyrin (Tretter et al., 2008, 2011; Mukherjee et al., 2011). On the other hand, GABA_A_R composed of α4, α5 or α6 subunits, together with β and δ subunits, are predominantly extrasynaptic, mediate tonic inhibition resulting mainly from synaptic “spillover” and are insensitive to benzodiazepine modulation (Brünig et al., 2002; Glykys and Mody, 2007; Jacob et al., 2008). The tonic inhibition in CA1 and CA3 pyramidal neurons is mediated by α5 and δ subunit-containing GABA_A_R (Glykys and Mody, 2006) that detect low, ambient concentrations of GABA in the extracellular space and desensitize slowly. Accordingly, deletion of the α5 subunit eliminates about half of the tonic currents mediated by GABA_A_R in hippocampal CA1 and CA3 pyramidal neurons; the remaining current was found to be mediated by GABA_A_R containing δ subunits (Glykys and Mody, 2006). Moreover, studies using mice bearing a point mutation in position 105 of the GABA_A_R α5 subunit, which downregulates the expression of the receptors exclusively in hippocampal pyramidal neurons, showed an important role for these subunits in cognitive processes (Crestani et al., 2002).

Under normal physiological conditions GABA_A_R respond to the binding of GABA by opening an integral chloride channel and allowing chloride to enter the neuron. The result is a membrane hyperpolarization and neuronal inhibition. This mechanism of inhibition by GABA_A_R depends on the electrochemical potential for chloride. Therefore changes of the intracellular Cl^−^ concentration ([Cl^−^]_i_) may regulate the response to the activation of GABA_A_R (Jedlicka et al., 2011). For example, in immature neurons GABA_A_R are mostly excitatory due to the fact that the intracellular chloride concentration is above the equilibrium. Maturation of the CNS is accompanied by a decrease of neuronal [Cl^−^]_i_, which accounts for the hyperpolarizing effect of the receptor (Watanabe and Fukuda, 2015).

Neuronal [Cl^−^]_i_ is mostly regulated by two chloride cotransporters, KCC2 (K^+^-Cl^−^ cotransporter; KCC type 2) and NKCC1 (the Na^+^-K^+^-2Cl^−^ cotransporter type 1; Russell, 2000; Blaesse et al., 2009). KCC2 expression is neuronal specific and under normal physiological conditions the transporter extrudes Cl^−^ out of the cell. NKCC1 is present in a variety of cells and generally loads cells with Cl^−^. Furthermore, the relative expression pattern of the two transporters differs across development (Russell, 2000; Ben-Ari, 2002). The NKCC1 transporter is more expressed earlier in development than KCC2, and this accounts for the high [Cl^−^]_i_ observed in immature neurons. In the mature brain, the increased abundance of KCC2 contributes to a lower [Cl^−^]_i_ when compared with the extracellular concentration, favoring the influx of Cl^−^ through the GABA_A_R channel and consequent membrane hyperpolarization upon activation of the receptors (Kaila et al., 2014).

The activity of GABA_A_R is also regulated by “cross-talk” with other receptors (Shrivastava et al., 2011a). Since GABA_A_R can be found in heterologous synapses (Nusser et al., 1996; Renner et al., 2012; de Luca et al., 2017), such receptor cross-talk may be mediated by a direct interaction with other receptors or through activation of intracellular signaling pathways. For example, GABA_A_Rs have been demonstrated to heteromerize with GABA_B_R (Balasubramanian et al., 2004), dopamine D5 receptors (Liu et al., 2000), purinergic P2X receptors (Jo et al., 2011; Shrivastava et al., 2011b), nicotinic acetylcholine receptors (Lee et al., 2010) and adenosine A_1_ receptors (Hu and Li, 1997). In particular, the cross-talk between GABA_B_R/GABA_A_R may contribute to their regulation at pre- and postsynaptic levels. For instance, a direct interaction of GABA_B1_ subunits with γ2S subunits of GABA_A_R was observed in the rat brain, and co-expression of GABA_B1_ subunits with GABA_A_R increases the inhibitory responses mediated by the latter receptors (Balasubramanian et al., 2004). Of particular interest is the NMDA receptor (NMDAR) mediated modulation of GABA_A_R. It has been demonstrated that activation of NMDAR downregulates GABA_A_R function due to calcium dependent activation of phosphatase 2B/calcineurin followed by dephosphorylation of GABA_A_R (Stelzer and Shi, 1994; Chen and Wong, 1995; Marsden et al., 2007; Bannai et al., 2009). A recent study showed that GABA_A_Rs are trapped at glutamatergic synapses in response to glutamatergic stimulation, thereby limiting GABA_A_R inter-synaptic diffusion (de Luca et al., 2017). The evidence that a hetero-synaptic interaction is modulated by neuronal activity suggests that cross-talk between GABA_A_R and other receptors may be considered a mechanism for tuning inhibition in the CNS.

**Figure 1:**
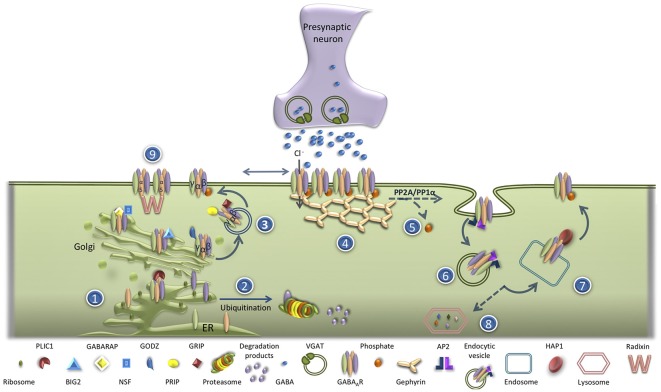
GABA_A_ receptor (GABA_A_R) trafficking under physiologic condition. (1) GABA_A_R are assembled in the ER. (2) In the ER, unassembled receptor subunits are subjected to poly-ubiquitination and targeted for proteasomal degradation. (3) GABA_A_R transport to the Golgi is a process negatively regulated by Plic-1. Inside the Golgi, GABA_A_R bind to GABA_A_R associated protein (GABARAP)/N-ethylmaleimide-sensitive factor (NSF) complex that facilitates their transport to the plasma membrane. The delivery of GABA_A_R to the plasma membrane is also regulated by GODZ, Big2, glutamate receptor-interacting protein (GRIP) and PRIP. (4) At the plasma membrane, GABA_A_R quickly exchange between synaptic and extrasynaptic locations, and the accumulation of the receptor at the inhibitory synapses is regulated by its scaffold protein gephyrin. (5) The phosphorylation of β3 or γ2 GABA_A_R subunits on their intracellular loop negatively regulates GABA_A_R internalization. (6) The process of GABA_A_R endocytosis is AP2/clathrin/dynamin-mediated. (7) Most internalized GABA_A_R are rapidly recycled back to the plasma membrane by a mechanism dependent of the interaction with huntingtin-associated protein 1 (HAP1). (8) The non-recycled GABA_A_R are targeted for lysosomal degradation.

Deficits in the functional expression of GABA_A_R have been implicated in the pathogenesis of several neurological and psychiatric diseases (Schwartz-Bloom and Sah, 2001; Rudolph and Knoflach, 2011; Kaila et al., 2014). GABA_A_R are assembled within the endoplasmic (ER) and are then transported to the Golgi. In the ER, unassembled receptor subunits are subjected to poly-ubiquitination that targets them for proteasomal degradation (Kittler et al., 2002), a phenomenon that is dependent on the level of neuronal activity (Saliba et al., 2007). This process is negatively regulated by Plic-1 (the protein that links integrin-associated protein with the cytoskeleton-1; Bedford et al., 2001), which binds directly to the α- and β-subunits of the receptor, prolonging their residence times in the ER (Figure 1). Inside the Golgi, GABA_A_R receptors bind to GABA_A_R associated protein (GABARAP)/N-ethylmaleimide-sensitive factor (NSF) complexes, facilitating their transport to the plasma membrane (Leil et al., 2004). This mechanism mediates the increase in the exocytosis of GABA_A_R observed upon stimulation of cultured hippocampal neurons with N-Methyl-D-aspartate (NMDA; Marsden et al., 2007). The delivery of GABA_A_R to the plasma membrane is regulated by Golgi-specific DHHC (Asp-His-His-Cys) zinc finger protein (GODZ), a Golgi resident palmitoyltransferase responsible for the palmitoylation of γ subunits. GODZ interacts with the GABA_A_R γ2 subunit recognizing a 14-amino acid cysteine-rich domain conserved in the intracellular domain of γ1–3 subunits, NH_2_-terminal to the GABARAP binding site (Rathenberg et al., 2004). The γ2 subunit is palmitoylated at all four cysteines within the GODZ binding domain (Rathenberg et al., 2004; Vithlani et al., 2011). The ADP ribosylation factor (Arf) guanine nucleotide exchange factor (GEF) Big2 (brefeldin A-inhibited GDP/GTP exchange factor 2) also plays a role in the delivery of GABA_A_R from the Golgi to the plasma membrane by promoting the budding and trafficking of vesicles from this compartment (Charych et al., 2004b). This protein interacts with the intracellular loop of all GABA_A_R β2 subunits (Charych et al., 2004b). Additional proteins important in the trafficking of GABA_A_R from the Golgi to the plasma membrane are the glutamate receptor-interacting protein (GRIP; Charych et al., 2004b; Kittler et al., 2004a), the phospholipase C-related catalytically inactive proteins 1 and 2 (PRIP1/2; Kanematsu et al., 2002; Uji et al., 2002), the GABA_A_R-interacting factor (GRIF-1; Beck et al., 2002) and Maf1 interacting coiled-coil protein (Macoco; Smith et al., 2010). The insertion into the membrane of the vesicles containing GABA_A_R also depends on SNAP23-syntaxin1A/B-VAMP2 complexes (Gu et al., 2016).

Once in the membrane, GABA_A_R are very dynamic, exchanging between synaptic and extrasynaptic locations (Jacob et al., 2005; Thomas et al., 2005; Bogdanov et al., 2006), being the accumulation of the receptors at the inhibitory synapses regulated by the scaffold protein gephyrin (Fritschy et al., 2008; Tyagarajan and Fritschy, 2014). Gephyrin recruitment to inhibitory synapses is a fundamental phenomenon for their long-term potentiation (iLTP). Studies using a chemical protocol to induce iLTP in cultured hippocampal neurons, consisting in a moderate activation of NMDARs, showed an increased synaptic clustering of GABA_A_R by a mechanism involving a CaMKII-dependent phosphorylation of GABA_A_R β3 subunits on S383 (Petrini et al., 2014). Potentiation of inhibitory synapses in the same model was found to be mediated by recruitment of gephyrin from extrasynaptic regions, downstream of GABA_A_R phosphorylation, as shown by single-particle tracking (SPT) analysis (Petrini et al., 2014). Recent studies using single-molecule super-resolution imaging with a novel clustering analysis, showed a rearrangement of synaptic gephyrin molecules during iLTP, with the formation of gephyrin nanodomains within the synaptic area (Pennacchietti et al., 2017).

GABA_A_R are in a continuous cycle between the plasma membrane and the intracellular compartments (Jacob et al., 2008; Mele et al., 2016). Regulation of the total GABA_A_R surface expression plays a key role in the control of the postsynaptic receptor pool size and the strength of synaptic inhibition (Mele et al., 2016). The process of GABA_A_R endocytosis occurs mainly *via* clathrin- and dynamin-dependent mechanisms upon interaction of GABA_A_R β and γ subunits with the adaptor protein 2 (AP2) clathrin adaptor protein complex (Kittler et al., 2000, 2005, 2008). In the brain, GABA_A_R interact with AP2 through a direct binding of the β1–3 and γ2 GABA_A_R subunits (Kittler et al., 2000). The first sequence motif important for AP2/clathrin/dynamin-mediated endocytosis of GABA_A_R was identified in an heterologous system and corresponds to a di-leucine motif present in β subunits (Herring et al., 2003, 2005). Additional studies performed in neurons, identified an amino acid sequence motif (KTHLRRRSSQLK in the β3 subunit), which includes a major phosphorylation site conserved in the cytoplasmic loop region of β1–3 subunits (Ser^408^, Ser^409^ in β3), as an important motif for AP2/clathrin/dynamin-mediated GABA_A_R internalization (Kittler et al., 2005, 2008). This motif also contains the major sites of phosphorylation by cAMP-dependent protein kinase A (PKA) and calcium/phospholipid-dependent PKC within this class of receptor subunits: Ser^409^ in β1, Ser^410^ in β2, and Ser^408/9^ in β3 (McDonald et al., 1998; Brandon et al., 2002, 2003; Kittler et al., 2005; Smith et al., 2008). Furthermore, a sequence of three arginine residues (^405^RRR^407^) was identified within the β3 subunit that is responsible for the interaction of GABA_A_R with AP2 and in the stabilization of the receptors at dendritic endocytic zones where they are internalized (Smith et al., 2012). The GABA_A_R internalization rate is negatively regulated by phosphorylation of β3 or γ2 GABA_A_R subunits on their intracellular loop. Thus, NMDAR signaling is known to control the stability of synaptic GABA_A_R *via* calcineurin-mediated dephosphorylation of the receptors (Muir et al., 2010). Moreover, a tyrosine-based AP2-μ2 adaptin-binding motif (Y^365^GY^367^ECL) was identified in the GABA_A_R γ2 subunit, which is also conserved in the γ1 and γ3 subunits (Moss et al., 1995; Kittler et al., 2008). These tyrosine residues are the major sites for phosphorylation by Fyn and Src kinases (Nishikawa et al., 2002; Jacob et al., 2005; Bogdanov et al., 2006), and their phosphorylation reduces AP2 binding (Kittler et al., 2008).

The internalized GABA_A_R may be rapidly recycled back to the neuronal plasma membrane or targeted for lysosomal degradation. The destiny of receptors following endocytosis is determinant for the regulation of surface/synaptic receptor abundance. The interaction of GABA_A_R β1–3 subunits with huntingtin-associated protein 1 (HAP1) determines whether endocytosed GABA_A_R are recycled (Kittler et al., 2004b). HAP1 is a GABA_A_R associated protein that binds the intracellular loop of β subunits *in vitro* and *in vivo* (Kittler et al., 2004b). Overexpression of HAP1 in neurons inhibits GABA_A_R degradation and consequently increases receptor recycling (Kittler et al., 2004b). Furthermore, HAP1 overexpression was shown to increase surface levels of GABA_A_R and miniature inhibitory postsynaptic current (mIPSC) amplitude in cultured hippocampal neurons (Kittler et al., 2004b).

**Figure 2:**
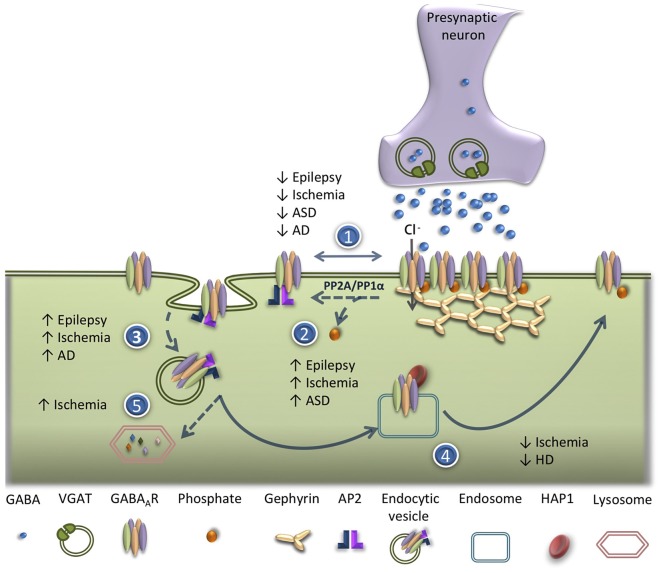
Alterations of GABA_A_R trafficking in brain disorders. Deficits in GABA_A_R trafficking have been reported in different pathological conditions in the central nervous system (CNS). (1) Reduced synaptic clustering of GABA_A_R has been observed in epilepsy, ischemia, autism spectrum disorders (ASDs) and Alzheimer’s disease (AD). (2) Increased dephosphorylation of GABA_A_R β3 subunit on serine residues 408/9 (Ser^408/409^) has been reported in epilepsy, ischemic condition and ASD. (3) An increase in AP2/clathrin/dynamin-mediated endocytosis of GABA_A_R occurs in epileptic conditions, ischemia, ASD and AD. (4) Impairment in GABA_A_R recycling has been shown in ischemic conditions and in Huntington’s disease (HD). (5) Enhanced lysosomal degradation of GABA_A_R due to ubiquitination was detected after an ischemic insult.

The balance between the insertion, lateral diffusion, internalization and recycling of GABA_A_R in the neuronal plasma membrane determines the strength of GABAergic synapses. Defects in GABA_A_R trafficking have been reported as triggers of GABAergic dysfunction in a number of brain pathological conditions (Hines et al., 2012). The following sections will address the alterations in GABA_A_R trafficking, in acute brain disorders, as well as in neuropsychiatric and neurodegenerative diseases (Figure 2).

## Alterations in the Rate of Constitutive Degradation and on the Trafficking of GABA_A_R in Epilepsy

Epilepsy is a chronic disorder of the brain characterized by the presence of recurrent spontaneous seizures. The disease affects approximately 65 million people worldwide, from all ages and both genders (Jacobs et al., 2009; Hesdorffer et al., 2013). In temporal lobe epilepsy, the most common form of partial epilepsy in humans, an initial insult is followed by a seizure-free period before the development of spontaneous seizures. The process by which the brain become hyperexcitable and prone to generate seizures is defined as epileptogenesis (Sharma et al., 2007; Curia et al., 2008). During the latent (seizure-free) period there is a complex reorganization of neuronal networks, which has been characterized in more detail in the hippocampus (Goldberg and Coulter, 2013). An increase in neuronal excitability may contribute to the genesis and/or propagation of epileptic seizures, and several cellular and molecular changes are thought to be involved in the development of spontaneous seizures following a brain insult (Loscher and Brandt, 2010; Goldberg and Coulter, 2013; Staley, 2015).

Studies in animal models have shown that the pathophysiology related with the appearance of seizures is associated with a dysfunction of GABAergic neurotransmission (El-Hassar et al., 2007). Accordingly, several antiepileptic drugs act as agonists of GABA_A_R (Czuczwar and Patsalos, 2001) and a dysfunction of GABA_A_R has been proposed to be involved in the etiology of epilepsy. In fact, mutations or genetic variants of the genes encoding the α1, α6, β2, β3, γ2, or δ subunits have been associated with human epilepsy (reviewed by Hirose, 2014). Also, mutations in GABA_A_R that enhance the constitutive ER-associated degradation (ERAD) of the receptors have been associated with genetically determined epilepsies, as well as, with idiopathic generalized epilepsies (Cossette et al., 2002; Huang et al., 2014). Furthermore, multiple GABA_A_R mutations associated with epilepsy result in the abnormal trafficking of the receptors (Kang et al., 2015), perturbing their expression on the plasma membrane and synaptic clustering (Han et al., 2015; Huang et al., 2017; Ishii et al., 2017).

Among genetic epilepsies displaying abnormal GABAergic neurotransmission, a group of pediatric monogenic epilepsies was characterized in patients with the Dravet and Rett syndromes (Ali Rodriguez et al., 2018; Gataullina et al., 2019). These disorders are associated with neurodevelopmental complications, and autism spectrum disorders (ASD)-like features are common in patients with both syndromes, suggesting a link between epilepsy and ASD (Ali Rodriguez et al., 2018). In fact, epilepsy is quite common in patients with ASD and therefore the association between epilepsy and autism is receiving growing interest (Deykin and MacMahon, 1979; Olsson et al., 1988; Galanopoulou et al., 2000; Giovanardi Rossi et al., 2000; Besag, 2004; Hughes and Melyn, 2005; Kosinovsky et al., 2005). In addition to the most common mutation in the SCN1A gene affecting the α1 subunit of voltage-gated sodium channels (Wu et al., 2015), Dravet syndrome may also result from mutations in genes that alter GABAergic transmission, such as GABRA1, GABRB2, GABRB3, and GABRG2, encoding the corresponding subunits of GABA_A_R (α1, β1, β2 and γ2 subunits, respectively). Moreover, a recent study identified a de novo heterozygous missense mutation in GPHN, which encodes for gephyrin, in a patient with Dravet-like syndrome (Dejanovic et al., 2017). Human mutations in the protocadherin-19 (PCDH19) gene, which encodes for the PCDH19 protein, also cause early infantile epileptic encephalopathy, associated with intellectual disability and autistic features (Kolc et al., 2019), similar to Dravet syndrome. PCDH19 cytoplasmic region binds to the α subunits of GABA_A_R thereby regulating the receptor surface expression, suggesting that PCDH19 might be involved in the regulation of GABA_A_R intracellular trafficking (Bassani et al., 2018). Furthermore, PCDH19 downregulation in hippocampal neurons causes a reduced frequency of mIPSCs (Bassani et al., 2018).

The primary cause of Rett syndrome is a mutation of the gene encoding the transcriptional repressor methyl-CpG-binding protein 2 (MeCP2; Kozinetz et al., 1993). Between 60 and 80% of females with Rett syndrome suffer from epilepsy (Vignoli et al., 2017). Studies performed in the Mecp2 KO animal model of Rett syndrome, showed a dramatic loss of GABAergic neurons (Chao et al., 2010). Moreover, recent evidence demonstrated that Mecp2 targets KCC2, and neurons differentiated from induced pluripotent stem cells from patients with Rett syndrome showed a reduced expression of KCC2 and a delayed switch in the excitatory to inhibitory responses to GABA during development (Tang et al., 2016).

The major problem in the therapy of status epilepticus (SE), and recurrent epileptiform discharges, is the time-dependent pharmacoresistance; about 30% of the patients become resistant to the treatment (Regesta and Tanganelli, 1999; French, 2007). A potential mechanism accounting for the impairment of inhibitory neurotransmission, characteristic of SE, and for the development of pharmacoresistance to benzodiazepines (De Koninck, 2007), is a reduction in the availability of functional GABA_A_Rs associated with the plasma membrane, which may arise from an altered pattern of receptor trafficking (Figure 2). Accordingly, *in vitro* studies performed in hippocampal neurons exposed to a medium lacking Mg^2+^, to induce epileptiform discharges, showed a reduction of about 50% in the surface expression of GABA_A_R after 1 h of SE, as demonstrated by a biotinylation assay (Cho et al., 2017). Furthermore, experiments using cultured hippocampal neurons incubated in a medium lacking Mg^2+^, an *in vitro* model of SE, showed a reduction in the surface stability of GABA_A_R as determined by live-cell imaging of SE pHluorin (SEP)-tagged α_2_ subunits. The observed decrease in the surface expression of GABA_A_R was mediated by activation of NMDARs for glutamate and was sensitive to inhibition of the phosphatase calcineurin (Eckel et al., 2015). Additional studies using the same *in vitro* model of SE combined with electrophysiological and cellular imaging techniques, showed that prolonged epileptiform bursting leads to a reduction of GABA-mediated synaptic inhibition; the constitutive internalization of GABA_A_R accelerated by the increased neuronal activity was associated with seizure activity. Moreover, inhibition of neuronal activity reduced the effect of SE on the rate of GABA_A_R internalization as well as the downstream reduction in the surface expression of the receptors that may contribute to the downregulation of inhibitory neurotransmission observed during seizures (Goodkin et al., 2005). This model is supported by evidence obtained in *in vivo* studies using the lithium-pilocarpine model of TLE, which showed a reduction in the amplitude of mIPSCs mediated by postsynaptic GABA_A_R when tested in dentate gyrus granule cells (Naylor et al., 2005). In contrast, the amplitude of extrasynaptic GABA_A_R tonic currents was increased during SE (Naylor et al., 2005). These results also suggests a possible increase in extracellular GABA concentration during SE, which may be coupled to an upregulation of extrasynaptic tonic currents, while synaptic currents may be decreased under the same conditions due to desensitization and internalization of GABA_A_R (Naylor et al., 2005). In fact, inhibition of GABA_A_R endocytosis in epileptic cultures resulted in both a recovery of the levels of membrane associated GABA_A_R and a total blockade of spontaneous recurrent epileptiform discharges (Blair et al., 2004).

In accordance with the role of GABA_A_R phosphorylation in the regulation of their surface expression (see above), SE reduces PKC-dependent phosphorylation of GABA_A_R β3 subunit on the serine residues 408/9 (Ser^408/409^; Terunuma et al., 2008). These residues contain a binding motif for the clathrin AP AP2, being a critical regulator of GABA_A_R endocytosis (Nakamura et al., 2015). Pharmacological activation of PKC or the specific blockade of GABA_A_R binding to AP2, during SE, restores the surface expression of the receptors, re-establishing the efficacy of synaptic inhibition (Terunuma et al., 2008).

The proper trafficking of GABA_A_R required to maintain the number and localization of the receptors at the neuronal surface is also dependent on the function of different proteins that interact with GABA_A_R directly or through adaptor proteins linked with microtubules (Mele et al., 2016). The expression of key scaffolding proteins associated with GABA_A_R is altered during epileptogenesis. For example, SE downregulates the expression of gephyrin and GRIP in the hippocampal CA1 region 4–8 days after the insult (pilocarpine injection; González et al., 2013). These alterations are correlated with changes in the plasma membrane expression and assembly of GABA_A_R (González et al., 2013). To what extent the downregulation of GRIP contributes to the observed reduction in the surface expression of GABA_A_R remains to be investigated. In fact, GRIP interacts with GABARAP (Kittler et al., 2004b) and is expressed at inhibitory postsynapses (Dong et al., 1999; Charych et al., 2004a; Li et al., 2005). Therefore, the SE-induced decrease in GRIP protein levels may impair the GABARAP-mediated delivery of GABA_A_R to the plasma membrane (Marsden et al., 2007).

Alterations in gephyrin clustering and expression during epileptogenesis were also detected in the hippocampus and in the cerebral cortex (Thind et al., 2010; Fang et al., 2011). The epileptogenic period is characterized by a reduction in the number of gephyrin puncta and GABAergic synapses in dentate gyrus, while an increased number of gephyrin clusters was detected during the chronic period (Thind et al., 2010). Moreover, studies performed in the neocortex showed that gephyrin expression gradually decreases during the epileptogenic period and returns to basal levels during the chronic phase (Fang et al., 2011). Thus, gephyrin downregulation may contribute to the instability of GABA_A_R clustering, amplifying the deficit in GABAergic neurotransmission observed in epileptic condition.

The ezrin/radixin/moesin (ERM) family protein radixin acts a scaffold to anchor α5βγ2 GABA_A_R to the actin cytoskeleton at extrasynaptic sites (Loebrich et al., 2006). This interaction is regulated by an activity-dependent manner through the RhoA-ROCK pathway (Hausrat et al., 2015). The dissociation of the receptors from the radixin anchor allows the lateral diffusion of GABA_A_R to increase their synaptic expression (Hausrat et al., 2015). However, whether this type of mechanism regulates the surface expression of GABA_A_R containing α5 subunits remains to be investigated.

Recent evidence indicates that alterations in chloride homeostasis may also contribute to the impairment of the GABA inhibitory activity (Rivera et al., 2004). These alterations have been attributed to a downregulation of the K^+^-Cl^−^ cotransporter KCC2. The resulting increase in the intracellular Cl^−^ concentration may account for the positive shift of the GABA_A_R reversal potential, and the consequent depolarizing effects of GABA, observed in hippocampal slices exposed to conditions mimicking status epilepticus (Coull et al., 2003). Interestingly, two independent studies reported that rare variants of KCC2 confer an increased risk of epilepsy in humans (Kahle et al., 2014; Puskarjov et al., 2014). However, whether the SE-induced alteration in GABA_A_R trafficking depends on the alteration in Cl^–^ gradient was not yet confirmed.

Taken together, the studies mentioned above indicate that during seizures, the persistent cell firing and GABA release may lead to the extracellular accumulation of GABA, causing desensitization and internalization of postsynaptic GABA_A_R. Moreover, alterations of scaffolding proteins associated with GABA_A_R, mainly gephyrin, contribute to the ultimate failure of inhibition observed in epilepsy. These mechanisms could account for the maintenance of recurrent seizure activity and benzodiazepine pharmacoresistance.

## A Decrease in GABA_A_R Anchoring at the Synapse and in Receptor Recycling Impair Inhibitory Synapses in Brain Ischemia

Cerebral ischemia is a pathological condition caused by insufficient blood supply to the brain, which leads to an increase in glutamatergic neurotransmission coupled to excitotoxic neuronal death. The down-regulation of GABAergic synapses in brain ischemia resulting from GABA_A_R desensitization (Gyenes et al., 1994) and a reduction of cell surface density of GABA_A_R (Nusser et al., 1997, 1998a), is one of the major factors contributing to excitotoxicity (Mele et al., 2014).

One of the first direct evidence suggesting that ischemic insults decrease the cell surface expression of GABA_A_R through an increase in receptor internalization (Figure 2) came from *in vitro* studies using ELISA, as a cell surface receptor assay (Mielke and Wang, 2005). These studies showed that transient incubation of cultured cortical neurons in the absence of oxygen and glucose to mimic global ischemia decreases cell surface GABA_A_R without altering the total expression of receptors. In fact, inhibition of receptor endocytosis with hypertonic sucrose treatment prevented receptor internalization. In the same study, the authors suggested that GABA_A_R internalization could contribute to neuronal death (Mielke and Wang, 2005). Similarly, studies using quantitative membrane protein biotinylation assays and immunocytochemistry confirmed that the abundance of plasma membrane-associated GABA_A_R was significantly decreased in cortical and hippocampal neurons exposed to oxygen and glucose deprivation (OGD). In this set of experiments the activation of phosphatidylinositol 3-kinase/Akt-dependent signaling pathway, through PTEN downregulation, was shown to protect neurons from the toxic effects of OGD by preventing the reduction in the surface expression of GABA_A_R (Smith et al., 2012). Results obtained with antibody feeding assay also showed that OGD-induces the internalization of GABA_A_R-α1 and β3 subunits in cultured hippocampal neurons by a dynamin-dependent mechanism (Mele et al., 2014). Additionally, it was reported that the down-modulation of GABA_A_R from dendritic clusters during OGD is dependent on the AP2 pathway for cell surface removal of the receptors. Moreover, blockade of this pathway reduced the neuronal death induced by OGD (Kittler et al., 2008).

The interaction between β3-subunit and AP2 seems to be critical for GABA_A_R reduction in synapses during ischemic insult (Smith et al., 2012). The identification of the intracellular domains (ICD) region of the β3-subunit that mediates the interaction with the clathrin adaptor AP2 also revealed the presence of three arginine residues (^405^RRR^407^) within this binding motif that are essential for the interaction with μ2–AP2; mutation of these residues impairs receptor recruitment to clathrin-coated pits, significantly reducing receptor endocytosis (Smith et al., 2012). Studies performed with a β3-subunit RRR motif mutant with a deficient AP2 binding site showed that the acute loss of synaptic GABA_A_R during OGD is mediated by an AP2/β3 interaction. Furthermore, blocking the internalization of GABA_A_R using a peptide competing with β3 for the binding to AP2 reduces OGD-induced cell death (Smith et al., 2012).

Interestingly, the β3-subunit RRR motif is located adjacent to a phosphorylation site, Ser^408^/Ser^409^, which is known to negatively regulate the internalization of the receptor when phosphorylated (Kittler et al., 2005, 2008). These phosphorylation sites are also regulated during an ischemic insult, both *in vivo* (using the transient middle cerebral artery occlusion—MCAO, a model of focal ischemia) and *in vitro* (OGD). In particular, it was found that brain ischemia induces the dephosphorylation of GABA_A_R β3-subunit (Ser^408^/Ser^409^) *in vitro* and *in vivo* (Mele et al., 2014). Studies with cultured hippocampal neurons subjected to OGD confirmed that the dephosphorylation of this domain is responsible for the observed increase in receptor internalization (Mele et al., 2014). Again, the consequent reduction in the surface expression of GABA_A_R was correlated with ischemia-induced cell death, since the transfection of hippocampal neuron with a phospho-mimetic mutant of GABA_A_R β3 subunit (SS^408/409^AA), which does not undergo internalization, reduced significantly the OGD-induced apoptotic neuronal death (Mele et al., 2014).

The destiny of GABA_A_R after endocytosis depends on their interaction with HAP1 (Kittler et al., 2004b). Under physiologic conditions most internalized GABA_A_R are rapidly recycled back to the plasma membrane, by a mechanism dependent of HAP1, while the remaining pool of receptors undergoes lysosomal degradation (Kittler et al., 2004b). Cultured hippocampal neurons subjected to OGD showed an impairment in receptor recycling that was correlated with a decrease in the interaction of the receptor with HAP1. This protein is indeed downregulated during OGD condition by a calpain mediated mechanism. When overexpressed, HAP1 protected hippocampal neurons from OGD-induced cell death (Mele et al., 2017).

The reported reduction in the number of synaptic GABA_A_R observed in brain ischemia may also be directly related with the ubiquitination-dependent degradation of the receptors. In particular the ubiquitination of lysine residues between amino acids 317–328 within the intracellular domain of the GABA_A_R γ2 subunit modulates the lysosomal targeting of the receptor. This process controls the efficacy of neuronal inhibition under basal conditions by regulating the accumulation of GABA_A_R at inhibitory synapses (Arancibia-Cárcamo et al., 2009). The deficit in neuronal inhibition under conditions of OGD also involves an enhanced degradation of GABA_A_R due to ubiquitination of a motif located within the intracellular domain of the γ2 subunit, with a consequent deficit in the cell surface stability of the receptors (Arancibia-Cárcamo et al., 2009).

Together, these studies point to postsynaptic alterations of GABAergic synapses as central players in synaptic dysfunction induced by brain ischemia. The internalization of GABA_A_R that accounts for the impairment in inhibitory neurotransmission may also be related with the synaptic instability of the receptor. Indeed, the gephyrin scaffold protein was found to be cleaved in cultured hippocampal neurons subjected to OGD, by a calpain-dependent mechanism. The resulting disassembly of the gephyrin lattice underneath the plasma membrane is likely to cause an inefficient synaptic anchoring of GABA_A_R (Costa et al., 2016). OGD also decreases GABA_A_R/gephyrin interaction, as shown in experiments of surface co-immunoprecipitation of GABA_A_R α1 subunits and gephyrin (Mele et al., 2014). The decrease in the interaction between GABA_A_R and its scaffold protein gephyrin suggests a possible alteration in the membrane dynamics of the receptor. An increased mobility of the receptors at the synapse may make them less confined within this compartment, and these receptors would become more prone to be internalized. However, further experiments are needed to better understand the alteration induced by ischemic insults on the lateral diffusion of GABA_A_R, and the signaling mechanisms involved, contributing to the impairment of GABAergic synapse strength. The internalization of GABA_A_R after an ischemic injury may explain, at least in part, the failure of receptor agonists or modulators in clinical trials for stroke (Amantea and Bagetta, 2017).

Alteration of the electrochemical gradient may also contribute to the impairment of GABAergic neurotransmission in brain ischemia. Several studies reported a decrease in KCC2 expression in brain ischemia (Galeffi et al., 2004; Papp et al., 2008; Jaenisch et al., 2010). Transient MCAO was found to decrease KCC2 mRNA levels, 1 day after reperfusion, and a consequent downregulation in the protein levels of the transporter was detected 7 days after reperfusion (Jaenisch et al., 2010). An attenuated expression of KCC2 in neurons subjected to an ischemic insult may trigger GABA-evoked depolarizing responses, thereby influencing plasticity and damage induced by stroke.

## Animal Models of Autism Spectrum Disorders (ASD) Are Characterized by a Downregulation of GABA_A_R and Alteration in Their Synaptic Distribution

ASD is a group of early-onset developmental disorders characterized by a variety of behavioral deficits and intellectual disability (Mattina et al., 2009). More than 80% of ASD cases are caused by genetic alterations (Rosenberg et al., 2009; Frazier et al., 2014; Baio et al., 2018). However, a huge number of genes have been identified associated to ASD, making difficult the study of the physiological pathways affected by these conditions.

The imbalance between neuronal excitation and inhibition within cortical circuits has been suggested as a cellular mechanism accounting for the behavioral and cognitive symptoms of ASD (Jenks and Volkers, 1992; Ramamoorthi and Lin, 2011; Yizhar et al., 2011). Although the neurobiological bases of ASD have not been clearly established, several genes related to autism were shown to encode synaptic proteins. Accordingly, an aberrant synaptic activity is characteristic of ASD patients (Howell and Smith, 2019). In particular, a dysfunction in the GABAergic system has been suggested to play an important role in the pathogenesis of ASD (Nielsen, 1990; Dhossche et al., 2002; Pizzarelli and Cherubini, 2011; Figure 2).

A recent study reported a decreased expression of membrane associated GABA_A_R-β3 subunits, as well as a downregulation of the phosphorylated form of the receptor subunit, in the sodium valproate (VPA)-induced rat model of ASD. The reduced phosphorylation levels of GABA_A_R-β3 subunit suggests alterations in the trafficking of the receptor, namely an increase in receptor internalization. The changes in GABAergic neurotransmission induced by prenatal exposure to VPA were also associated to impaired spatial memory, limited exploration, increased anxiety, and reduced sociability (Li et al., 2017b).

Alterations in the phosphorylation of GABA_A_R γ2 subunits may also be relevant for the ASD phenotype as shown in studies using the Ser^408/409^Ala homozygous mice, in which the receptor subunit shows a low interaction with the AP2 complex which decreases internalization, similarly to the behavior of phosphorylated receptors. These animals are characterized by an increase in the activity of synaptic GABA_A_R, together with a reduction in the extrasynaptic inhibitory currents, and exhibit the core phenotypes of ASD (Vien et al., 2015). The *fmr1* KO mice which are commonly used as a model to study the fragile X syndrome and ASD also display an increased phosphorylation of GABA_A_R γ2 on Ser^408/409^ (Vien et al., 2015), further pointing to a role for alterations in the phosphorylation state of this subunit in neuropsychiatric disorders.

Deficits in GABA_A_R surface expression were also detected in mice with a loss-of-function of *PX-RICS* that results in ASD-like behaviors (Nakamura et al., 2016). These mice recapitulate the pathogenic process of ASD-like behavior characteristic of Jacobsen syndrome (JBS) patients (Mattina et al., 2009). *PX-RICS*^−/−^ mice exhibit a dysfunction of the postsynaptic mechanism for GABA_A_R trafficking. Cell surface labeling and biotinylation assays revealed that GABA_A_R γ2 surface expression is significantly reduced in *PX-RICS*^−/−^ hippocampal neurons and in cerebellar granule neurons (CGNs). Moreover, whole-cell patch–clamp experiments detected a reduction in the amplitude of mIPSCs with no significant differences in their frequency, suggesting that the postsynaptic responsiveness to inhibitory input is impaired without alteration in the presynaptic release of the neurotransmitter. Interestingly, stimulation with a GABA_A_R agonist improved some autistic-like phenotypes of *PX-RICS*^−/−^ mice (Nakamura et al., 2016). This suggests that a potentiation of postsynaptic GABAergic signaling could be a possible therapeutic strategy for ASD-like behavior.

The impairment of GABAergic neurotransmission in patients with ASD is further supported by evidence showing increased levels of Hrd1 in the middle frontal cortex of patients with ASD (Crider et al., 2014). This E3 ligase ubiquitinates misfolded GABA_A_R α1 subunits before ERAD in HEK293 cells (Di et al., 2016). Interestingly, a downregulation of GABA_A_R α1 subunits was also detected in the middle frontal cortex of ASD patients (Crider et al., 2014).

Mutations in several proteins associated with the postsynaptic density (PSD) of excitatory synapses have been associated with neuropsychiatric disorders (Volk et al., 2015; Li et al., 2017a; Gandal et al., 2018). The growing interest in the characterization of the inhibitory PSD (Tyagarajan and Fritschy, 2014) may shed light into the complexity of the mechanisms involved in the regulation of GABAergic neurotransmission and may show novel molecular players involved in the regulation of the surface dynamics of GABA_A_R with a role in neuropsychiatric disorders, including ASD.

## GABA_A_-Receptor Trafficking Involvement in Neurodegenerative Disorders

Alterations in GABA_A_R trafficking coupled to the dysregulation of the synaptic excitatory/inhibitory balance are also a common feature of several neurodegenerative diseases, such as Alzheimer’s disease (AD), Parkinson’s disease (PD) and Huntington’s disease (HD; Figure 2). These alterations might induce changes in synaptic strength and ultimately lead to excitotoxicity and consequent neuronal cell death.

AD is a chronic and progressive neurodegenerative disease characterized by memory deficits and cognitive decline owing to synaptic and neuronal loss in the hippocampus and cerebral cortex. The abnormal deposition of amyloid-β (Aβ) in these brain regions suggests that this peptide plays an essential role in AD pathogenesis (Karran and De Strooper, 2016). In fact, the observed deleterious effects of Aβ were shown to arise, in part, from the interaction of the peptide with NMDAR, causing excitotoxity and neuronal dysfunction (Costa et al., 2012).

GABAergic signaling was also demonstrated to be profoundly altered in the AD brain (Limon et al., 2012). Indeed, GABA currents were shown to desensitize faster and the GABA_A_R were found to be less sensitive to GABA after micro-transplantation of membranes from the temporal cortex of AD patients into *Xenopus oocytes* (Limon et al., 2012). Aβ was also shown to weaken synaptic inhibition through downregulation of GABA_A_R *via* receptor endocytosis (Ulrich, 2015). Accordingly, Aβ induced a decline in mIPSCs in layer V pyramidal neurons, an effect that was prevented using an inhibitor of the dynamin-mediated internalization of GABA_A_R (Ulrich, 2015). This result indicates that the observed hyperexcitability characteristic of AD could be partly related with the loss of functional GABA_A_R observed in the AD brain (Limon et al., 2012) and with the loss of synaptic inhibitory strength induced by Aβ (Ulrich, 2015).

In the context of AD, GABA_A_R were also show to suffer several consistent alterations in their subunit composition (e.g., α1, α2, α5, β2, β3 and γ2), in different brain regions, namely in the hippocampus (Kwakowsky et al., 2018). The complexity of these alterations is not compatible with simple compensatory mechanisms, but may reflect instead the reorganization of defined neuronal circuits (Kwakowsky et al., 2018). Despite these results, the effects of Aβ on inhibitory synapses are still poorly understood as most studies have focused on the impairment of excitatory synaptic transmission. In particular, the signaling pathways by which Aβ induce GABA_A_R endocytosis remain to be investigated. Since Aβ enhances neuronal excitability though NMDA activation and synaptic plasticity (Parihar and Brewer, 2010; Costa et al., 2012; Varga et al., 2014), this may constitute the signal to induce the internalization of GABA_A_R. Future studies should also address a possible direct interaction of Aβ with GABA_A_R or with proteins associated with the inhibitory PSD. Whether the tau pathology in AD is also somehow related with alterations in GABA_A_R traffic also remains to be investigated. Furthermore, the implications of the alteration in GABA_A_R trafficking in AD progression are still unclear. Several studies suggested that part of the symptoms associated to this disorder might be caused by the loss of the synaptic excitatory/inhibitory balance (Michels and Moss, 2007; McDade et al., 2009; Ulrich, 2015).

Alterations in GABA_A_R trafficking have also been associated with PD. This long-term neurodegenerative disorder mainly affects the motor system and causes a characteristic combination of motor symptoms (e.g., hypertonia) due to progressive neurodegeneration of dopaminergic neurons (Gilbert et al., 2006; Meder et al., 2018). The symptomatic treatment of hypertonia can be achieved by enhancing GABAergic transmission. Indeed, the regulation of GABA_A_R homeostasis was reported to be disrupted in a hypertonic mouse model bearing a mutation in the *hyrt* gene, which codes for the trafficking protein kinesin binding 1 (Trak1). This study showed a marked reduction in the levels GABA_A_R in the CNS, particularly in the lower motor neurons, and, interestingly, Trak1 was found to interact with GABA_A_R (Gilbert et al., 2006). Trak1 (and Trak2) shares some homology with HAP1 (Li et al., 1995), which has been implicated in intracellular trafficking and transport of GABA_A_R (Kittler et al., 2004b; Gilbert et al., 2006). In contrast with the effect on the expression of GABA_A_R, the distribution of the GABA_A_R anchoring protein gephyrin was not altered in *hyrt* mice. Therefore, the reduction in GABA_A_R in *hyrt* mice may be due to the dysregulation of GABA_A_R endocytic trafficking rather than to the destabilization of the plasma membrane complex that stabilizes the receptors at the synapse (Gilbert et al., 2006). Thus, it can be hypothesized that Trak1 may facilitate the targeting of endocytosed receptors back to the membrane or it may block their degradation. Interestingly, no significant degeneration of GABAergic neurons was observed in *hyrt* mice despite the reduction in the levels of GABA_A_R subunits in this hypertonic mouse model (Gilbert et al., 2006), as described for AD (Ulrich, 2015).

Other proteins have been associated with the reduction of GABA_A_R surface expression in PD. GABARAPs are a family of proteins that play a role in vesicle and receptor trafficking (Kittler et al., 2001), and in particular they were shown to interact and regulate the intracellular trafficking of GABA_A_R (Wang et al., 1999; Chen et al., 2001; Chen and Olsen, 2007). Furthermore, members of this protein family have been implicated in autophagy (Rowland et al., 2006; Schwarten et al., 2009), a mechanism involved in GABA_A_R clearance (Rowland et al., 2006). A recent study showed that GABARAPs also bind the parkin-associated endothelin-like receptor (PAELR; Dutta et al., 2018), which is localized in the core of Lewy bodies, a PD hallmark (Murakami et al., 2004). Furthermore, PAELR interacts with the GABA_A_R binding site of GABARAPL2, and this protein together with Parkin and PICK1 are most likely involved in the regulation of PAELR protein levels. This occurs *via* autophagy, ubiquitination and proteasomal degradation (Dutta et al., 2018), which ultimately might lead to the regulation of GABA_A_R trafficking. However, additional studies are required to establish a role for GABARAPs in PD.

HD is an autosomal dominant progressive neurodegenerative disorder caused by the mutant huntingtin (Htt), with an expanded polyglutamine (polyQ) repeat (McClory et al., 2014). This disorder is characterized by progressive involuntary choreiform movements, emotional disturbances and cognitive decline (Pinborg et al., 2001), associated with degeneration of GABAergic neurons (Fritschy and Brünig, 2003).

An early study using emission tomography methods (PET and SPECT) showed a reduction in the abundance of benzodiazepine receptors in the striatum (but not in the cortex) of HD patients (Pinborg et al., 2001). These binding sites are present in GABA_A_R containing, for example, α1, α2, α3, or α5 subunits, together with β and γ subunits, and are mainly located at the synapse where they mediate most phasic inhibition in the brain (Jacob et al., 2008). This contrasts with the extrasynaptic GABA_A_R that mediate tonic inhibition, which are insensitive to benzodiazepines (Jacob et al., 2008). The putative alterations in the expression of GABA_A_R in HD requires further investigation since immunohistochemistry experiments showed an increase in the abundance of the α1 and γ2 receptor subunits in the globus pallidus of patients with the disease, while the levels of gephyrin were not changed (Thompson-Vest et al., 2003). The discrepancy between the results obtained in the analysis of benzodiazepine receptors and expression of GABA_A_R subunits may be due to differences in the brain regions analyzed, which was more restricted in the latter case.

In contrast with the evidence showing changes in the abundance of GABA_A_R in certain brain regions of HD patients, the alterations in receptor trafficking in the disease have been poorly investigated. As mentioned before, HAP1 interacts directly with GABA_A_R and regulates inhibitory synaptic transmission by modulating GABA_A_R recycling (Kittler et al., 2004b). GABA_A_R are trafficked to synapses by the kinesin family motor protein 5 (KIF5), which mediates the insertion of GABA_A_R into the plasma membrane, and HAP1, the adaptor that links the motor protein to the receptors. Accordingly, HAP1-KIF5 dependent GABA_A_R trafficking was reported as a fundamental mechanism controlling the strength of synaptic inhibition in the brain (Twelvetrees et al., 2010). Mutant huntingtin containing a polyQ expansion disrupts the HAP1-KIF5 GABA_A_R trafficking and synaptic delivery (Twelvetrees et al., 2010). Thus, the disruption of this complex by mutant huntingtin may lead to altered synaptic inhibition and increased neuronal excitability in HD (Twelvetrees et al., 2010).

The disruption of GABA_A_R trafficking and synaptic inhibition was also observed in a mouse model of HD (Yuen et al., 2012). In the latter study, GABA_A_R-mediated inhibitory transmission was found to be disrupted in the HD at the symptomatic stage, a consequence of a diminished surface GABA_A_R expression, which may underlie the impaired GABAergic transmission (Yuen et al., 2012). Furthermore, the KIF5-mediated microtubule-based transport of GABA_A_R was confirmed to be impaired in HD, which may underlie the disruption of GABA_A_R trafficking to the synaptic membrane. Therefore, the interference in the effect of polyQ-Htt on the HAP1/KIF5-mediated trafficking of GABA_A_R to synapses may constitute a therapeutic approach for HD, by restoring synaptic function (Yuen et al., 2012).

The chronic neuroinflammation observed in these neurodegenerative disorders induces the upregulation of tumor necrosis factor-α (TNF-α), which might play a role in the observed synaptic excitatory/inhibitory unbalance (Frankola et al., 2011). TNF-α was already described as an important mediator of homeostatic synaptic plasticity (Stellwagen and Malenka, 2006), and, interestingly, it was shown to modulate GABA_A_R trafficking, thereby downregulating the inhibitory neurotransmission. Indeed, TNF-α enhances the association of protein phosphatase 1 (PP1) with GABA_A_R β3 subunits and dephosphorylates the amino acid residue of the β3 subunit responsible for the regulation of the phospho-dependent interactions with the endocytic machinery (Pribiag and Stellwagen, 2013).

## Final Remarks

Aberrant excitability is a common feature of numerous disorders of the CNS. Dysfunction of GABAergic synapses and in particular alterations of postsynaptic GABA_A_R trafficking have been reported as a key mechanism that contributes to the unbalance between excitation an inhibition, which ultimately will lead to neuronal hyperexcitability. Interestingly, similar alterations in the mechanism coupled to an increased internalization of GABA_A_R result in distinct outcomes/symptoms associated to different pathologies of the CNS. Depending on the circuits, the brain region and the developmental stage in which the postsynaptic alteration of GABAergic system is initiated, different structural and molecular modifications of the involved neurons may occur, triggering distinct pathologic responses. However, the disruption of the GABAergic neurotransmission characteristic of various illnesses may partly account for some common symptoms. For example, patients with cerebral ischemia, as well as certain cases of ASD or HD (Gambardella et al., 2001), may present seizures that are a hallmark of epilepsy. The reviewed studies indicate that the mechanisms involved in the control of plasma membrane and synaptic expression of GABA_A_R are key players in the modulation of neuronal excitability. However, considering the recent findings showing that the nanoscale redistribution of the scaffold protein gephyrin is a key event in the potentiation of inhibitory synapses, additional studies are required to evaluate the alterations in GABA_A_R and gephyrin nanoscale redistribution induced by hyperexcitability in pathological conditions. The outcome of this type of studies may contribute to the identification of novel therapeutic targets for various brain disorders characterized by an impaired regulation of the excitation/inhibition balance.

## Author Contributions

MM, RC and CD wrote the manuscript.

## Conflict of Interest Statement

The authors declare that the research was conducted in the absence of any commercial or financial relationships that could be construed as a potential conflict of interest.
